# Characterization of a Goose-Origin Avian Orthoreovirus with Interferon Suppression Activity

**DOI:** 10.3390/v18040447

**Published:** 2026-04-08

**Authors:** Yijia Liu, Yong Li, Yingxuan Xie, Mei Wang, Boxuan Yin, Changyan Li, Lilin Zhang, Deping Hua, Junwei Liu, Xintian Zheng, Jinhai Huang

**Affiliations:** 1 School of Life Sciences, Tianjin University, Tianjin 300072, China; jiaawd@163.com (Y.L.); yong.li@tju.edu.cn (Y.L.); 18222718536@163.com (Y.X.); 2023226011@tju.edu.cn (M.W.); 2022226012@tju.edu.cn (B.Y.); lichangyanlcy@163.com (C.L.); lyliazhang@tju.edu.cn (L.Z.); huadeping@tju.edu.cn (D.H.); 2 Qingdao Runda Biotech Co., Qingdao 266200, China; lasota@126.com; 3 College of Life Sciences, Longyan University, Longyan 364012, China; xintianzheng@lyun.edu.cn

**Keywords:** *Avian orthoreovirus*, σC protein, host adaptation, innate immune response, transcriptome analysis

## Abstract

The emergence of variant strains of *Avian orthoreovirus* (ARV) has caused substantial economic losses in the poultry industry worldwide, but the molecular features of goose-origin strains and viral transmission among different avian species remain poorly understood. Here, we describe a goose-origin avian orthoreovirus strain, SD0407, associated with growth retardation and joint swelling. Complete genome analysis identified ten double-stranded RNA segments. Sequence comparison indicated that SD0407 is closely related to previously reported duck-origin reovirus strains. Phylogenetic and recombination analyses showed that most segments clustered with duck-origin strains, indicating close genetic relatedness among waterfowl-origin orthoreoviruses. Sequence and structural analysis of the σC attachment protein revealed ten unique amino acid substitutions, including D250 within the DE-loop region involved in receptor-binding. Molecular docking suggested that σC interacts with the conserved AnxA2-S100A10 heterotetrameric receptor complex, providing a possible structural basis for receptor compatibility across avian species. Although SD0407 replicated efficiently in goose embryo fibroblasts, it did not induce expression of type I, II or III interferons. Transcriptome profiling revealed weak activation of innate immune signaling and downregulation of metabolic and cytoskeletal genes, consistent with effective suppression of antiviral responses. These findings demonstrate that SD0407 combines structural variability with immune evasion to enhance host adaptability and underscore the importance of sustained ARV surveillance in waterfowl populations.

## 1. Introduction

*Avian orthoreovirus* (ARV) is a non-enveloped, segmented double-stranded RNA virus and a persistent threat to the global poultry industry. Classically, ARV is associated with viral arthritis/tenosynovitis and malabsorption syndrome, both of which cause significant morbidity, mortality, and economic losses in commercial broilers [1,2]. In recent years, the pathogenic spectrum of ARV has become more complex, with atypical strains linked to pericardial effusion-hepatitis syndrome, respiratory disease, and even neurological manifestations [3]. These emerging clinical forms complicate field diagnosis and underscore the importance of continuous molecular surveillance to capture viral diversity.

Genetic evolution analysis is central to understanding the emergence and spread of ARVs. As segmented double-stranded RNA viruses, ARVs evolve through point mutations, reassortment, and recombination [4]. These processes generate extensive genetic diversity, especially within the *S1* segment encoding the outer capsid protein Sigma C (σC), a key determinant of host tropism and antigenicity [5,6]. Recent studies have shown that waterfowl-origin ARVs frequently cluster apart from chicken strains, reflecting distinct evolutionary trajectories [7,8]. Moreover, reassortment between lineages has been documented, producing recombinant viruses with altered pathogenicity and host range [9]. Such genetic plasticity highlights the capacity of ARVs to adapt rapidly under immune and ecological pressures. Despite these insights, the evolutionary dynamics of goose-origin ARVs remain largely unexplored. Comprehensive phylogenetic and recombination analyses are therefore required to clarify their origins, evolutionary relationships, and potential role in cross-species transmission.

Among structural proteins, the σC plays a central role in viral biology. It mediates host cell attachment, determines tissue tropism, and serves as the main target of neutralizing antibodies [5,6,10]. However, the *S1* gene encoding σC displays extreme sequence divergence, particularly within its C-terminal receptor-binding region, showing up to 46% nucleotide and 53% amino acid variation among field and vaccine isolates [11,12]. Antigenic drift within σC contributes directly to vaccine failures, which have been increasingly reported across Asia, Europe, and North America [13]. Studies have shown that even single amino acid substitutions within the σC receptor-binding domain can significantly alter antigenicity and cross-neutralization [14]. Consequently, σC not only represents the principal determinant of host interaction but also a molecular marker of viral evolution and immune escape. Understanding σC variability is thus essential for predicting ARV emergence and guiding the rational design of next-generation vaccines.

Traditionally considered pathogens of chickens, avian orthoreoviruses are now increasingly recognized in waterfowl. Novel strains from ducks and geese have been reported in China, Vietnam, and Europe, with some causing severe necrotizing hepatitis, splenic necrosis, and growth retardation [15,16,17,18]. Comparative genomics has revealed substantial divergence between waterfowl- and chicken-origin strains, suggesting independent evolutionary lineages [13]. Even more concerning, reassortment between chicken- and waterfowl-origin ARVs has been documented, generating recombinant viruses with expanded host range and enhanced virulence [8,17,19,20,21]. These findings highlight the role of waterfowl as both reservoirs and amplifiers of novel ARV variants. However, while duck ARVs have received growing attention, goose-origin ARVs remain poorly studied. The scarcity of genomic data, pathogenicity studies, and antigenic analyses of goose strains represents a critical gap in ARV research.

In addition to genomic diversity, ARVs have evolved multiple molecular strategies to modulate host innate immune responses. Recent studies have revealed that several viral proteins target distinct steps of the RIG-I-like receptor (RLR) and interferon (IFN) signaling pathways. The σA protein interacts directly with interferon regulatory factor 7 (IRF7), the functional homolog of IRF3 in birds, and prevents its dimerization and nuclear translocation, thereby suppressing type I IFN transcription [22]. The multifunctional p17 protein modulates host immunity through dual mechanisms. It can bind to the Hsp90/Cdc37 chaperone complex to regulate NF-κB signaling and chaperone-mediated autophagy, while also inhibiting activation of the RIG-I signaling pathway, which results in reduced interferon production [23]. In addition, the σNS protein contributes to immune evasion by forming viral factories that sequester cytoplasmic signaling molecules and prevent their interaction with downstream adaptors such as MAVS [24].

Similar immune evasion strategies have been described in mammalian orthoreoviruses (MRVs), where viral uncoating kinetics influence immune sensing. Rapidly uncoating MRV strains can escape from endosomal detection and thereby reduce RLR-mediated interferon activation [25]. These findings suggest that orthoreoviruses employ conserved but diverse mechanisms to suppress interferon-mediated antiviral responses. However, whether goose-origin ARVs possess comparable immunosuppressive capabilities remains largely unknown. Understanding how SD0407 interacts with the host interferon system is therefore essential for clarifying its immune evasion strategy and cross-species adaptability.

Therefore, the primary aim of this study was to isolate and comprehensively characterize a goose-origin avian orthoreovirus strain, SD0407 and to conduct integrated genomic, structural, and transcriptomic analyses. Particular attention was given to σC-mediated receptor binding and the modulation of host antiviral responses. By elucidating the unique σC variations and host antiviral responses associated with SD0407 infection, this study seeks to provide new molecular insight into the evolution and host interaction of waterfowl-origin avian orthoreoviruses.

## 2. Materials and Methods

### 2.1. Virus Isolation and Propagation

The ARV strain SD0407 was recovered in our laboratory from spleen samples of diseased domestic geese (*Anser cygnoides*) exhibiting growth retardation and joint swelling in Shandong Province, China. Spleen tissues were homogenized, and the clarified supernatant was collected for virus isolation. The original clinical samples were screened by PCR/RT-PCR for other major avian pathogens, including avian influenza virus, Newcastle disease virus, and waterfowl parvoviruses. In addition, a σC gene fragment was amplified from the original material and subjected to sequence analysis. The clarified supernatant was inoculated into the allantoic cavity of 13-day-old goose embryos for virus isolation. For embryo inoculation experiments, 9-day-old SPF chicken embryos and 10-day-old duck embryos were used. After 48–72 h, the allantoic fluid was harvested, clarified, and stored at −80 °C. Viral RNA was extracted from the allantoic fluid using RNAiso Plus reagent (Takara Bio Inc., Shiga, Japan). The isolate was subsequently identified by segment-specific RT-PCR amplification and sequencing of all 10 genome segments. Primary goose embryo fibroblasts (GEFs) and duck embryo fibroblasts (DEFs) were prepared from 9–12-day-old embryos and cultured in DMEM (Gibco, Grand Island, NY, USA) containing 10% fetal bovine serum at 37 °C with 5% CO_2_. GEFs were infected with SD0407 to evaluate viral replication and cytopathic effects (CPEs). Viral replication kinetics were quantified by real-time qPCR targeting the S4 gene. The relative viral RNA expression levels were calculated using the 2^−ΔΔCt^ method, normalized to the host internal reference gene (GAPDH) [26]. Clarified allantoic fluid from infected avian embryos was examined by transmission electron microscopy using a Hitachi TEM system at 80 kV.

### 2.2. Viral RNA Extraction, Segment-Specific RT-PCR Amplification, and Genome Sequencing

Viral RNA was extracted from 200 μL of infected allantoic fluid using the RNAiso Plus (Takara Bio Inc., Shiga, Japan) reagent according to the manufacturer’s instructions. Ten pairs of segment-specific primers were designed based on conserved regions of avian orthoreovirus reference strains available in GenBank (Table S1), and used to amplify all 10 double-stranded RNA genomic segments via reverse transcription-polymerase chain reaction (RT-PCR).

First-strand cDNA synthesis was performed using the Hifair^®^ III 1st Strand cDNA Synthesis Kit (gDNA digester plus) (YEASEN, Shanghai, China) with gene-specific primers. PCR products were purified using a Gel Extraction Kit (Novoprotein, Shanghai, China), ligated into the pTOPO-T vector (GenStar, Beijing, China), and transformed into *Escherichia coli* DH5α competent cells. Positive clones were selected and subjected to bidirectional Sanger sequencing, which was performed commercially by Tsingke Biological Technology Co., Ltd. (Beijing, China).

### 2.3. Bioinformatics and Phylogenetic Analysis

The complete viral genome sequences of all 10 segments were obtained from segment-specific RT-PCR products and annotated using standard bioinformatics workflows. Sequence similarity searches were performed against the GenBank database using the web-based BLAST (https://blast.ncbi.nlm.nih.gov/, accessed on 6 November 2025) at the National Center for Biotechnology Information (NCBI) [27]. All reference strains and their corresponding citations used in this study are listed in Supplementary Table S3 [20,28,29,30,31,32,33,34,35,36,37,38,39,40,41,42,43,44,45,46]. Multiple sequence alignments of nucleotide sequences and their deduced amino acid sequences were conducted in MEGA11 using the built-in ClustalW algorithm with default parameters. Phylogenetic trees were constructed based on amino acid sequences using the neighbor-joining (NJ) method implemented in MEGA11 with the Poisson substitution model and 1000 bootstrap replicates to evaluate branch support. The resulting trees were visualized and edited using the Interactive Tree of Life (iTOL)v7.5 (https://itol.embl.de/, accessed on 6 December 2025). Potential recombination events were detected using RDP4 and SimPlot (v3.5.1) with default settings. Only recombination signals supported by at least three detection methods in RDP4 were considered significant.

### 2.4. Analysis of the Structure and Function of σC

The amino acid sequence of σC was retrieved from the reference strain. Secondary structure was predicted using JPred4 web server (University of Dundee, Dundee, UK) [47] with default parameters. The three-dimensional structure was modeled using AlphaFold3 (DeepMind Technologies Limited, London, UK) [48], and the highest confidence model was selected. Functional motifs, including receptor-binding regions, were analyzed by sequence alignment and by comparison with published data. Specific amino acid substitutions relative to the reference strain were mapped onto the 3D model.

The host receptor complex (Annexin A2/S100A10 heterotetramer) was modeled based on the human crystal structure (https://www.rcsb.org/structure/1BT6, accessed on 24 December 2025). The goose Annexin A2 sequence was retrieved from GenBank and structurally aligned with the human AnxA2 using PyMOL 2.0 (Schrödinger, LLC, New York, NY, USA) [49]. The human AnxA2 chains were subsequently replaced with the goose model to generate a species-specific AnxA2-S100A10 heterotetramer, which was used for molecular docking with the SD0407 σC trimer. Molecular docking was performed using HADDOCK 2.4 web server (https://wenmr.science.uu.nl/haddock2.4/, Utrecht University, Utrecht, The Netherlands) accessed on 6 January 2026 [50] and validated by the ClusPro server (https://cluspro.org, Boston University, Boston, MA, USA) accessed on 6 January 2026 [51], focusing on predicted receptor-binding regions. Docking results were ranked by score, and the top complexes were visually inspected. Structural visualization and interface analysis were performed using PyMOL 2.0.

### 2.5. RNA-Seq Analysis and Quantitative Real-Time PCR Validation

Total RNA was extracted from mock- and SD0407-infected goose embryo fibroblast (GEF) cells at 24 h post-infection using RNAiso Plus reagent. High-quality RNA was sequenced by Novogene (Beijing, China) on the Illumina NovaSeq 6000 (Illumina, San Diego, CA, USA) platform to generate 150 bp paired-end reads. The Virus and Mock samples generated 43.61–46.89 million raw reads, corresponding to 6.54–7.03 Gb of raw bases. After quality control using fastp v0.20.1, 42.46–45.66 million clean reads (6.37–6.85 Gb) were retained for downstream analysis. The Q20 and Q30 values ranged from 98.88% to 99.25% and 97.24% to 98.51%, respectively. Clean reads were mapped to the goose reference genome (*Anser cygnoides*, GCF_000971095.1) with HISAT2 v2.0.5, and the corresponding annotation file for the same genome assembly was used for read counting and downstream analysis. Gene expression levels were calculated as FPKM using featureCounts v1.6.3, and differential expression was analyzed with edgeR R package (3.22.5). Genes with |log_2_FC| ≥ 1 and adjusted *p* < 0.05 were considered differentially expressed. Heatmap and volcano plots were plotted by https://www.bioinformatics.com.cn (last accessed on 10 December 2024), an online platform for data analysis and visualization. For validation, representative genes (Table S2) were quantified by qRT-PCR using Hieff^®^ SYBR Green Master Mix (YEASEN, Shanghai, China). Relative expression was calculated by the 2^−ΔΔCt^ method with GAPDH as the reference gene.

## 3. Results

### 3.1. Virus Isolation, Phenotypic Characterization, and Genome Identification

**Figure 1:**
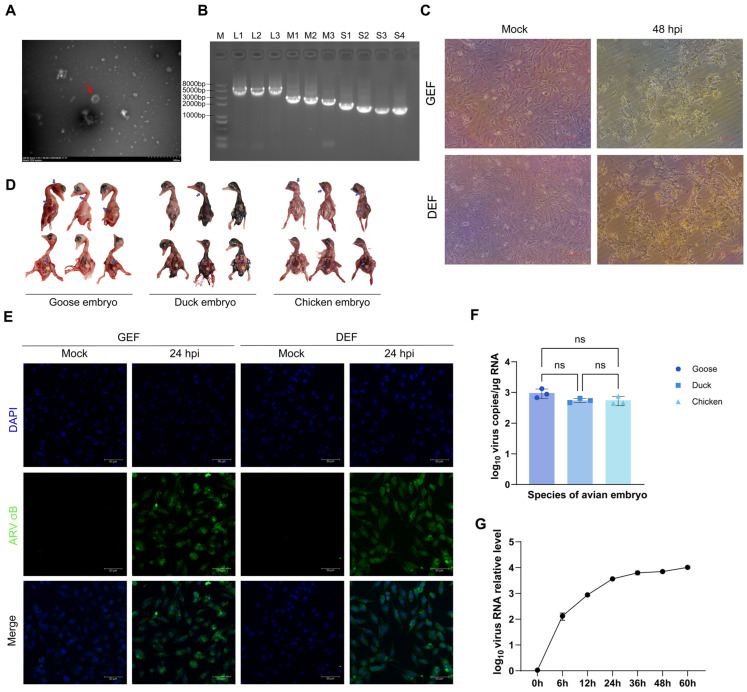
Isolation and characterization of the goose-origin avian orthoreovirus strain SD0407. (**A**) Transmission electron microscopy showing spherical virus particles (~80 nm in diameter; red arrow), consistent with typical orthoreovirus morphology. (**B**) Agarose gel electrophoresis of the ten double-stranded RNA genomic segments of SD0407. (**C**) Cytopathic effects (CPE) in primary goose embryo fibroblasts (GEFs) and duck embryo fibroblasts (DEFs) at 24 h post-infection (hpi), showing characteristic syncytium formation in infected cells. Images were acquired at 20× magnification. (**D**) Representative gross lesions in goose, duck, and chicken embryos inoculated with SD0407, including subcutaneous hemorrhage and hepatic necrosis. Blue arrows indicate hemorrhagic foci. (**E**) Immunofluorescence detection of viral σB protein (green) in infected GEFs and DEFs at 24 hpi using anti-σB antibody; nuclei were counterstained with DAPI (blue). (**F**) Absolute quantitative PCR (qPCR) of viral RNA in allantoic fluid from infected goose, duck, and chicken embryos, showing no significant difference among species. (**G**) Growth kinetics of SD0407 in GEFs determined by qPCR, with viral RNA levels plateauing from 36 hpi and reached the highest level at 60 hpi. All quantitative data are presented as mean ± standard deviation (SD) from three independent experiments. Statistical significance was determined using one-way ANOVA followed by Tukey’s multiple comparison test (*p* < 0.05); ns, not significant.

The goose-origin orthoreovirus strain SD0407 was isolated from spleen tissue samples of diseased geese by inoculation into avian embryos. Other major avian pathogens were excluded by PCR/RT-PCR screening before virus isolation. Its biological characteristics were then evaluated. No clear CPE was observed in untreated control DEF and GEF cells. In contrast, after three successive passages, infected cells exhibited marked CPE, and typical syncytial formation was evident at 48 h post-infection (hpi) (Figure 1C). Inoculated embryos also developed hemorrhage and hepatic lesions (Figure 1D). To evaluate replication kinetics, SD0407 was propagated in primary GEF cells, and viral titers were quantified at multiple time points post-infection by qRT-PCR targeting the S4 gene. The viral growth curve showed that viral RNA levels increased progressively after infection, entered a plateau phase from 36 hpi, and reached the highest level at 60 hpi (Figure 1G), consistent with the typical replication dynamics of avian orthoreoviruses [52]. To assess viral replication in different embryos, viral RNA was extracted from infected allantoic fluid and detected by qRT-PCR. All three embryo types tested positive for the S4 gene, with no significant differences in viral copy number (Figure 1F).

To further verify infection, an immunofluorescence assay (IFA) was performed using a mouse polyclonal antibody raised against the SD0407 σB protein. Strong cytoplasmic fluorescence signals were observed in infected GEF and DEF cells at 24 hpi, whereas mock-infected controls showed no signal (Figure 1E), confirming efficient viral replication and antigen expression in host cells.

Furthermore, transmission electron microscopy of clarified allantoic fluid from infected embryos revealed spherical viral particles with a diameter of approximately 80 nm, consistent with typical orthoreovirus morphology (Figure 1A).

### 3.2. Phylogenetic Analysis of the Viral Genome

The complete genome of a goose-origin reovirus was determined (Table S1). The genome consists of 10 segments, ranging from 1156 to 3959 bp (Figure 1B). It should be noted that the genome was amplified utilizing primers targeting the universally conserved 5′ (GCUUUU) and 3′ (UCAUC) termini typical of ARVs (Table S1); thus, these terminal motifs in our final assembled sequences are intrinsically primer-derived. Each segment contains an intact ORF encoding either structural or non-structural proteins. Major structural proteins include core proteins (λA, λB, λC, μA, σA) and outer capsid proteins (μB, σB, σC), responsible for genome packaging, RNA polymerase activity, and cell entry. Non-structural proteins (NS, P10, P17) are involved in replication, assembly, and adsorption. These genomic features are consistent with the typical segmented nature of reoviruses.

**Figure 2:**
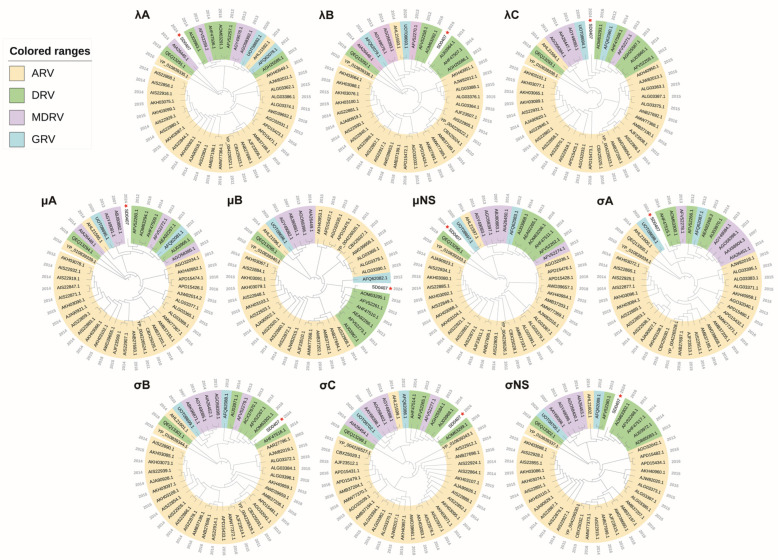
Phylogenetic analysis of the viral genome. Phylogenetic trees constructed using the neighbor-joining method in MEGA11 based on 10 viral proteins (λA, λB, λC, μA, μB, μNS, σA, σB, σC, σNS) The SD0407 strain is indicated by a red pentagram. In this classification, ARV refers to chicken-/turkey-origin avian orthoreoviruses, whereas DRV, MDRV, and GRV refer to duck-, Muscovy duck-, and goose-origin orthoreoviruses, respectively.

To further analyze the genetic evolution of the SD0407 strain, phylogenetic analysis of the amino acid sequences of 10 proteins from various avian reovirus strains was performed using MEGA11 software. As shown in Figure 2, the σC phylogenetic tree revealed that the SD0407 strain clustered with duck reovirus strains.

In the λA and λB phylogenetic trees, the SD0407 isolate clustered in the same clade as the Duck reovirus strain SD-12 (λA: AIJ03963.1, λB: AIJ03964.1) [42], which was consistent with the sequence alignment results.The phylogenetic trees of λC, μB, σC, and σNS proteins showed that the SD0407 isolate clustered with the Duck reovirus strain HN5d (λC: AOM63293.1, μB: AOM63295.1, σC: AOM63299.1, σNS: AOM63302.1), which was in line with the sequence alignment results.In the σA and σB phylogenetic trees, the SD0407 isolate clustered in the same clade as the Duck reovirus strain ZJ00M (σA: AHF47515.1, σB: AHF47516.1) [44], consistent with the sequence alignment results.The μA and μNS proteins of SD0407 clustered in the same clades as those of Duck reovirus strain 091 (μA: AFV52260.1) [36] and Goose reovirus (GRV)-GD2020 (μNS: UOT08697), respectively. Notably, μNS was the only one among the 10 proteins that clustered with goose reovirus.

In the phylogenetic trees (Figure 2), our isolate SD0407 (2024) was clustered within a clade whose origin could be traced back to the 2014–2018 period. This clade contained multiple avian reovirus strains isolated during 2014–2018, and SD0407 showed a close phylogenetic relationship with these strains isolated in the earlier decade, forming a monophyletic group with them in the tree.

### 3.3. Genomic Sequence Identity and Recombination Analysis

**Figure 3:**
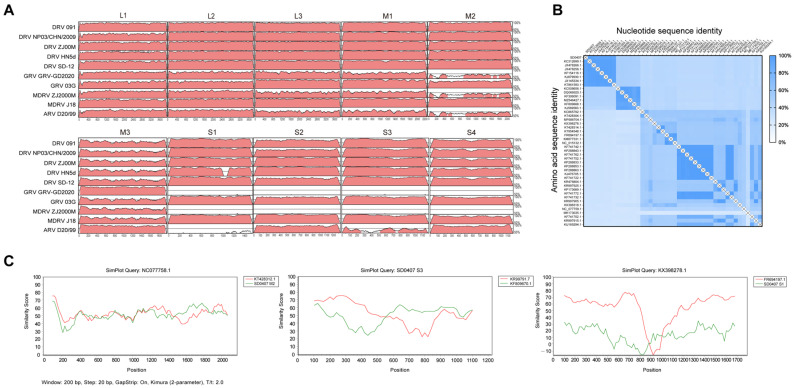
Genomic sequence identity and recombination analysis. (**A**) Heatmap of genomic sequence identity between strain SD0407 and 10 reference strains visualized using the mVISTA platform; red regions indicate nucleotide sequence similarity ranging from 50% to 90% (**B**) Heatmap of nucleotide and amino acid sequence identity of the major antigenic protein σC between SD0407 and reference strains (**C**) SimPlot-based recombination analysis with sequences showing more than three recombination events in RDP4 validated by SimPlot and crossover regions indicating potential recombination events.

To compare the complete genome of SD0407 with other reference strains, ten representative strains with high sequence identity were selected, and genome-wide analysis was performed using the mVISTA (https://genome.lbl.gov/vista/mvista/submit.shtml, accessed on 6 January 2026) platform. As shown in Figure 3A, SD0407 does not share uniformly high nucleotide similarity with any single reference strain. Instead, it exhibits a distinct mosaic similarity pattern across different segments with several waterfowl-origin strains (such as DRV NP03/CHN/2009, DRV ZJ00M, and GRV-GD2020) [30,44], strongly suggesting potential inter-segmental reassortment. Importantly, the classical strain ARV D20/99 [32] displayed markedly lower sequence identity in the *S1* segment encoding the attachment protein σC when compared with the other reference strains, suggesting that vaccines derived from classical strains may provide reduced protection against this goose-origin strain. Recombination analysis of SD0407 was further conducted using SimPlot (window size: 200 bp, step size: 20 bp, Kimura two-parameter model). In the *M2* segment, SD0407 showed moderate to high similarity (scores 40–70) with KT428312.1 [38] across the 1200–2000 bp region. In the *S1* segment, a sharp decrease in similarity with FR694197.1 [28] was observed around 900 bp, with negative values detected in certain regions, indicating potential genetic divergence or recombination. In the *S3* segment, SD0407 exhibited a distinct recombination crossover between putative parental strains KR997917.7 [33] and KF809670.1 [32] at approximately 550 bp, reflecting complex genetic relationships and recombination signals (Figure 3C). These results suggest that multiple segments of SD0407 may have undergone recombination, providing valuable clues for understanding ARV evolution and cross-species transmission, and offering new insights into the genetic adaptation of this strain.

### 3.4. Sequence and Structural Analysis of the σC Protein

The σC protein serves as the primary viral attachment protein, mediating cell binding and neutralizing antibody responses. Comparative sequence analysis across strains revealed substantial heterogeneity, with both nucleotide and amino acid identities ranging from approximately 22% to 100%. Waterfowl and chicken ARV strains shared under 50% identity, forming distinct phylogenetic clusters (Figure 3B). SD0407 showed the highest σC similarity with Duck reovirus NP03/CHN/2009 (96.26% amino acid, 96.48% nucleotide), matching its clustering in phylogenetic analysis. In contrast, the lowest similarity occurred with Duck reovirus strain Ych [29] (22.57% amino acid, 38.98% nucleotide), which lies in a separate phylogenetic branch.

**Figure 4:**
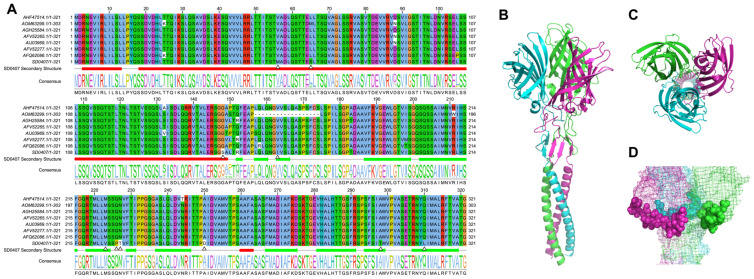
Sequence and structural analysis of the σC protein. (**A**) Multiple sequence alignment of σC amino acid sequences from SD0407 and nine closely related strains with >90% identity; white triangles indicate amino acid residues unique to SD0407 (**B**,**C**) Predicted three-dimensional structure of σC protein from strain SD0407 (**D**) Mapping of the DE-loop region onto the σC protein structure.

Alignment of SD0407 σC with eight highly homologous DRV reference strains revealed ten unique amino acid substitutions (M56, S65, S148, I163, V223, P226, T227, D250, T298, L310; Figure 4A). These mutations, particularly at residue D250 within the putative DE-loop receptor-binding region (approx. aa 244–267), may influence host receptor interaction and cross-species transmission potential.

To explore structural implications, we used AlphaFold to predict the SD0407 σC trimer. The model features a β-barrel globular head and an N-terminal α-helical stalk (coiled-coil), consistent with known structures of ARV S1133 σC and mammalian reovirus σ1-responsible for receptor binding-particularly in their head domain topology (Figure 4A,B). The presence of the DE-loop motif within the globular domain reinforces its role in receptor engagement. Notably, the substitution at position 250 may alter the receptor-binding interface, suggesting a potential mechanism for altered host specificity or antigenicity (Figure 4C).

### 3.5. Structural and Functional Analysis of σC Reveals Antigenic Variability and Conserved Receptor-Binding Interface

**Figure 5:**
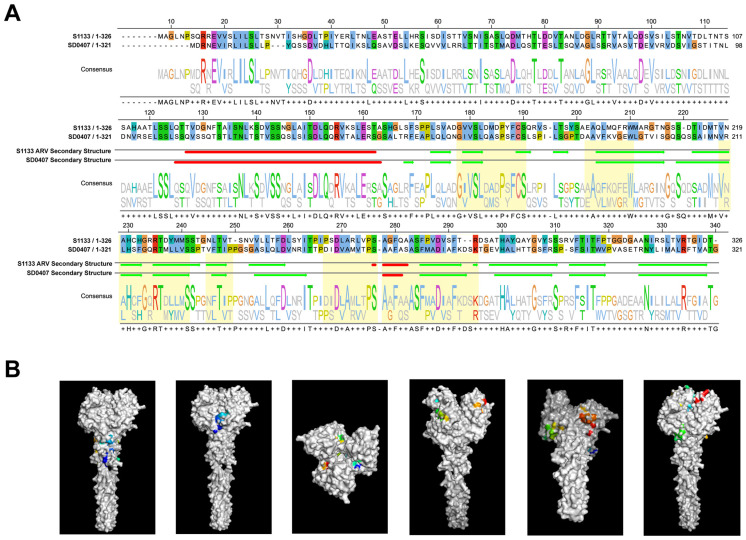
Antigenic epitope analysis of the σC protein (**A**) Comparison of the σC protein from strain SD0407 with that of the avian reovirus reference strain S1133 with previously reported antigenic peptide sequences highlighted in yellow (**B**) Spatial distribution of the first six antigenic peptides mapped onto the protein surface with colored regions indicating their corresponding positions.

To further evaluate the antigenic properties of the SD0407 strain, the predicted linear B-cell epitopes of ARV S1133 σC were mapped onto the σC sequence of SD0407 (Figure 5A, highlighted in yellow). Comparative analysis revealed that SD0407 contained multiple amino acid substitutions within the predicted epitope regions, while several key residues remained conserved. This indicates that although SD0407 σC exhibits notable sequence divergence from S1133, certain antigenic determinants are evolutionarily preserved.

To assess the structural relevance of these epitopes, we mapped the predicted antigenic peptides onto the three-dimensional structure of SD0407 σC (Figure 5B). All six predicted epitopes were exposed on the surface of the trimeric protein, consistent with their potential accessibility to host antibodies. Mutations within these epitopes may alter local surface topology, thereby affecting antibody recognition or receptor-binding affinity. This structural observation suggests a possible link between σC variability and host interaction or antigenic variation.

Given that σC also mediates viral attachment to host cells, we next examined its potential interaction with host receptor proteins. Previous studies have suggested that annexin A2 (AnxA2) serves as a receptor for avian reoviruses (ARVs) [53]. AnxA2 is a Ca^2+^-dependent phospholipid-binding protein that plays roles in membrane trafficking, endocytosis, and virus entry. In host cells, AnxA2 often forms a heterotetramer complex with S100A10, which stabilizes its membrane association and facilitates protein-protein interactions [54].

**Figure 6:**
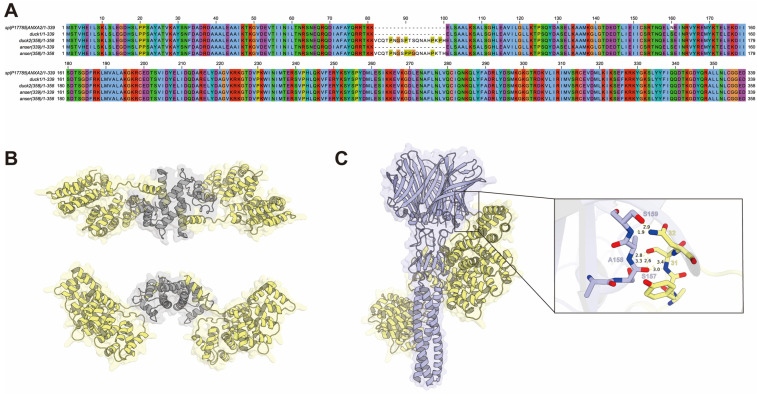
Molecular docking of σC with the host receptor (**A**) Alignment of ANXA2 amino acid sequences from chicken, duck, and goose (**B**) Predicted structure of the ANXA2-S100A10 heterotetramer (**C**) Docking prediction of the SD0407 with the ANXA2-S100A10 receptor complex showing the highest-confidence model and adjacent interacting residues. Yellow dashed lines indicate polar interactions, and the numbers represent interatomic distances in Å.

To investigate potential receptor compatibility across avian species, we compared AnxA2 amino acid sequences from chicken, duck, and goose (Figure 6A). Sequence alignment revealed a very high identity (99.8%), suggesting that conserved receptor structure may underlie the ability of ARVs to cross avian species barriers. To further explore the interaction between SD0407 σC and host receptors, we modeled the AnxA2-S100A10 heterotetramer based on available PDB templates and performed molecular docking with the predicted σC trimer (Figure 6B). Docking analysis suggested that residues within the σC DE loop (260–263 aa) may interact with the receptor (Figure 6C). These results highlight the DE loop as a critical region mediating host receptor binding, consistent with its proposed role in receptor recognition in orthoreoviruses.

Together, these results highlight the structural plasticity of σC in the goose-origin ARV strain SD0407: conserved residues within the DE loop ensure receptor-binding capacity across avian hosts, whereas variable surface epitopes facilitate antigenic drift and potential immune escape. Such dual functionality provides a mechanistic explanation for how σC contributes simultaneously to host adaptation and cross-species transmission.

### 3.6. SD0407 Infection Fails to Activate the Interferon Response and Suppresses the IFN Signaling Pathway

**Figure 7:**
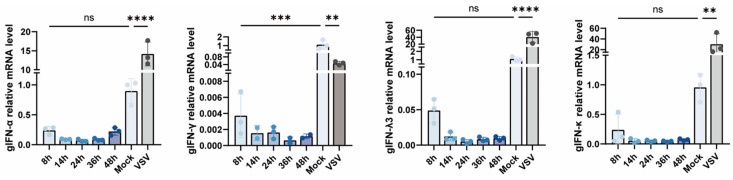
The qRT-PCR analyses reveal that SD0407 infection does not induce interferon expression in GEF cells. Expression of interferon genes (IFN-α, IFN-κ, IFN-λ3, IFN-γ) at different time points post infection detected by qRT-PCR. SD0407 infection failed to induce interferon expression, while VSV served as a positive control. Data are presented as mean ± SD. Asterisks indicate statistical significance: ns, not significant; ** *p* < 0.01, *** *p* < 0.001, and **** *p* < 0.0001.

To investigate whether SD0407 infection triggers host antiviral responses, the transcriptional levels of type I and III interferons (*gIFN-α*, *gIFN-γ*, *gIFN-κ*, and *gIFN-λ3*) were examined in infected goose embryo fibroblasts (GEFs) (Figure 7). The results showed that interferon expression remained unchanged at all time points post-infection, whereas the positive control virus (VSV) induced a marked upregulation of interferon transcripts. These findings indicate that, despite efficient replication in host cells, SD0407 failed to elicit a typical interferon response.Previous studies have demonstrated that avian orthoreoviruses suppress interferon signaling through diverse mechanisms. The p17 protein interferes with IFI16-mediated sensing [55], σA binds IRF7 to block its activation [22], and μNS sequesters IRF3 within viral factories [56]. These mechanisms collectively disrupt IFN induction at multiple levels, suggesting that SD0407 may adopt a similar strategy to evade host antiviral responses.

Transcriptomic analysis revealed no marked activation of antiviral or interferon-related genes following SD0407 infection (Supplementary Figure S1B). Core molecules involved in the IFN response, including IFN receptors (*IFNGR2*, *IFNAR1*), JAK–STAT components (*STAT1*, *STAT2*), interferon-stimulated genes (*OASL*, *MX*, *IFIT5*), and innate immune sensors (*RIG-I*, *IRF7*), showed statistically significant but low expression levels. To confirm this, selected genes were further evaluated by qRT-PCR (Supplementary Figure S1B). Consistent with the transcriptomic findings, no statistically significant differences were observed between the infected and control groups, indicating that SD0407 fails to elicit a robust interferon response at the transcriptional level.

### 3.7. Transcriptomic Profiling Reveals Suppression of Host Immune Signaling After SD0407 Infection

**Figure 8:**
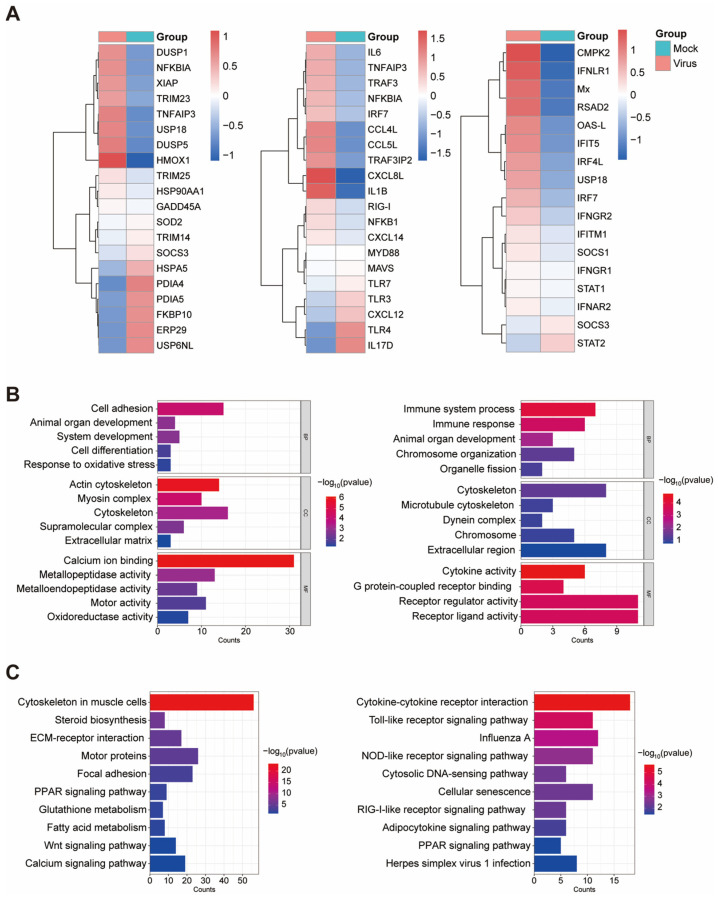
Differential gene expression and pathway enrichment analyses of SD0407-infected GEF cells. (**A**) Heatmaps showing differential expression of representative genes related to immune signaling (**left**), cytokine response (**middle**), and interferon or stress-associated pathways (**right**) between mock- and SD0407-infected GEF cells. Red and blue indicate up- and downregulation, respectively. (**B**) Gene Ontology (GO) enrichment analysis of upregulated and downregulated genes in three categories: biological process (BP), cellular component (CC), and molecular function (MF). The x-axis indicates the number of genes enriched in each GO term, and color represents −log_10_(*p*-value). (**C**) Kyoto Encyclopedia of Genes and Genomes (KEGG) pathway enrichment of upregulated (**left**) and downregulated (**right**) genes. Bars are colored according to −log_10_(*p*-value). These results indicate that SD0407 infection affects immune-related signaling, cytoskeletal organization, and multiple metabolic pathways in GEF cells.

To further elucidate the host transcriptional response to SD0407 infection, differential expression and enrichment analyses were conducted between infected and control cells. Hierarchical clustering revealed distinct transcriptional remodeling (Figure 8A). Upregulated genes, including IL6, TNFAIP3, TRAF3, and IRF7, were predominantly associated with inflammatory and cellular stress responses, whereas downregulated genes such as HSPA5, SOD2, and PDIA4 were linked to protein folding and oxidative homeostasis, suggesting virus-induced endoplasmic reticulum and oxidative stress.

GO and KEGG enrichment analyses (Figure 8B,C) showed that upregulated genes were modestly enriched in innate immune and cytokine-related pathways, including Toll-like, NOD-like, and RIG-I-like receptor signaling, indicating limited activation of antiviral sensing. In contrast, downregulated genes were mainly involved in metabolic and cytoskeletal organization pathways, such as glutathione metabolism, steroid biosynthesis, and calcium signaling, implying disruption of cellular metabolism and structural maintenance.

Overall, these results demonstrate that while SD0407 infection triggers broad transcriptional alterations, it elicits only a weak activation of interferon and antiviral signaling pathways. This transcriptional pattern underscores the virus’s ability to dampen host immune recognition and promote immune evasion, potentially facilitating its persistence and cross-species adaptation.

## 4. Discussion

To date, studies on ARVs have mainly focused on chicken- and duck-origin strains, with extensive reports on their pathogenicity, molecular evolution, and cross-species potential [1,3]. In contrast, goose-origin ARVs remain poorly characterized, and current literature lacks experimental or clinical evidence demonstrating their ability to infect heterologous avian hosts such as ducks or chickens [19,32,57]. Previous reports have only described the genetic relatedness of goose isolates to duck or chicken ARVs, but without functional confirmation of host range [8,58]. Therefore, the present study provides additional data on goose-origin avian orthoreovirus strains and supports further investigation of their host range and evolution. This represents a new perspective on the ecology and evolution of ARVs and highlights the importance of monitoring goose-origin strains in the broader context of avian reovirus epidemiology.

SD0407 (2024, goose-origin) clusters within the 2012–2022 clade, which contains chicken-, duck-, and goose-origin reoviruses. This finding provides insights into the genetic relationship of this viral lineage across different avian hosts. Notably, goose-origin reoviruses in this clade were isolated as late as 2022. Our SD0407 strain from goose (2024) shares close phylogenetic affinity with this multi-host viral group. This suggests that the clade contains closely related avian orthoreovirus strains from different avian hosts. The relatively late isolation of goose-origin strains in the clade (2022 onwards, including SD0407 in 2024) further implies geese may be a recent host for this lineage. This likely results from interspecies transmission from chicken or duck hosts earlier in the clade’s evolution. Notably, the clade’s division into two subclades in the μB protein tree and SD0407’s clustering with duck reoviruses and GRVs hint at possible genetic divergence within the lineage. Such host-associated clustering emphasizes the need for enhanced surveillance of avian reoviruses across poultry species. It may promote the emergence of strains with expanded host ranges and potential pathogenic risks. Although this study highlights the close genetic relatedness of ARVs, the precise temporal signals and directionality of interspecies spillover were not investigated. Such phylodynamic analyses are currently limited by historical sampling biases in waterfowl, representing an important area for future research.

Recombination analysis of strain SD0407 revealed potential genetic exchange among different genome segments, providing important clues for understanding the evolution of waterfowl-origin avian orthoreoviruses. The *M2* segment showed moderate to high sequence similarity with KT428312.1, suggesting a possible genetic relationship and shared evolutionary history, which may help preserve conserved structural functions. In contrast, the *S1* segment (encoding the σC protein, a key factor in host interaction) displayed a sharp drop in similarity around 900 bp, indicating genetic divergence or recombination. Such events may introduce additional genetic variations that contribute to host adaptation, which is particularly relevant to the unexplored issue of cross-species infection of goose-origin ARVs. The *S3* segment also exhibited complex genetic relationships and recombination signals, supporting the notion of genome reassortment that may contribute to the genetic diversity of SD0407. Taken together, these findings highlight the genetic plasticity of ARVs and underscore the need to investigate how recombination shapes viral adaptability, providing new insights into the ecology of avian orthoreoviruses.

σC is the major attachment protein of ARV and a key determinant of host range and antigenicity. Sequence comparison showed that SD0407 harbors several unique amino acid substitutions, including D250 within the DE-loop of the globular domain. The DE-loop is a recognized receptor-binding region in both avian and mammalian orthoreoviruses, mediating the first step of cell attachment [10]. Mutations in this region may alter binding affinity or broaden receptor usage. Thus, the D250 substitution could contribute to host adaptation and cross-species transmission.

Previous work demonstrated that even single-residue changes in the receptor-binding domain of mammalian reovirus σ1 can markedly affect receptor specificity and host range [59]. Similar observations in avian reoviruses suggest that structural variation in σC, especially within the DE-loop, may underlie the ability of certain strains to infect waterfowl hosts beyond their typical reservoir. The relatively high identity of SD0407 with duck-origin strains supports this hypothesis, while divergence from other DRVs highlights ongoing evolutionary pressures. In addition, σC variation may influence immune recognition. The DE-loop is exposed and can serve as a neutralizing epitope. Mutations here may enhance immune escape while preserving receptor binding [12]. This dual role could accelerate viral adaptation when ARV crosses between avian species. Taken together, the unique substitution at residue 250 in SD0407 highlights the importance of σC structural plasticity. It may represent a molecular signature for host switching and immune evasion, warranting further functional studies using reverse genetics and receptor-binding assays.

The σC protein is the major attachment protein of ARV and a key target of neutralizing antibodies. Our analysis showed multiple mutations in predicted antigenic peptides of SD0407 compared with the classical strain S1133, while several amino acid residues remained conserved. Mutations within antigenic regions may alter epitope conformation and reduce antibody recognition, which has been linked to immune escape and vaccine failure in ARV [60]. The persistence of conserved residues suggests functional constraints, likely related to protein stability or receptor binding. Structural mapping revealed that all predicted antigenic sites are located on the protein surface, consistent with their accessibility to host antibodies. Mutations clustered on these surface regions highlight the potential for altered antigenicity. Similar findings have been reported in waterfowl-origin ARVs, where σC variability correlates with cross-species transmission [13]. Thus, the observed mutations in SD0407 may not only influence immune recognition but also contribute to host adaptation. While these genetic variations suggest current vaccines may provide limited protection, developing goose-specific vaccines faces significant challenges: the high mutation dynamics of σC risk rapid immune escape, ARV genotypes do not strictly correlate with serotypes, and widespread vaccination is economically impractical in the limited-scale goose industry. Therefore, identifying these variants primarily underscores the critical need for continuous epidemiological surveillance to monitor antigenic drift and potential cross-species spillover to the broader poultry industry. Future studies employing reverse genetics and receptor-binding assays are warranted to experimentally elucidate how the σC protein balances immune evasion via surface epitope mutations with the conservation of host receptor affinity.

Our docking analysis suggests that the DE loop of SD0407 σC directly interacts with the host receptor complex AnxA2-S100A10. AnxA2 has been identified as a cofactor for ARV entry and replication, and its high sequence conservation among avian species likely facilitates cross-species transmission [53,61]. The observed binding at residues 260–263 is consistent with prior findings that flexible loops within reovirus attachment proteins mediate receptor recognition and host adaptation [62]. Mutations within this region may therefore alter binding affinity, potentially enhancing viral fitness in new avian hosts. These findings provide structural evidence that conserved AnxA2 receptors could serve as a molecular basis for ARV host switching. Moreover, they highlight the σC DE loop as a potential hotspot for adaptive evolution. Future studies should validate these interactions experimentally and assess their role in cross-species infection.

Transcriptomic and functional analyses revealed that SD0407 infection failed to induce a robust interferon response, despite efficient replication. Both type I and III interferons, as well as key interferon-stimulated genes (*STAT1*, *IRF7*, *MX*, *OASL*), remained weakly expressed, suggesting potent viral suppression of interferon signaling. Similar mechanisms have been reported for other avian orthoreoviruses. SD0407 may employ comparable strategies, possibly through direct interference with host transcription factors or signaling adaptors [22,55,56,63].

Consistent with this, transcriptomic enrichment showed only mild activation of innate immune and cytokine pathways, accompanied by downregulation of metabolic and cytoskeletal genes (*HSPA5*, *SOD2*, *PDIA4*). This pattern indicates that SD0407 reprograms cellular metabolism and stress responses while dampening antiviral gene activation. Such combined effects may create a cellular environment favorable for viral persistence [64]. These findings highlight the ability of SD0407 to evade host immune recognition and suppress interferon-mediated antiviral defenses, providing mechanistic insight into its immune evasion. Whether SD0407 evades the host immune response by passively shielding viral RNA or actively antagonizing interferon signaling pathways remains to be fully elucidated. Future investigations employing exogenous immune stimulants, such as poly(I:C) or recombinant goose interferon, in infected cells will be crucial to experimentally differentiate between these immune evasion strategies.

In conclusion, this study successfully isolated and comprehensively characterized a goose-origin avian orthoreovirus strain, SD0407, which is genetically closely related to previously reported duck-origin reoviruses. Our integrated analyses establish that specific variations in the σC protein, particularly within the DE-loop, confer structural adaptability for receptor recognition. Furthermore, transcriptomic profiling provides definitive evidence that SD0407 actively evades host immunity by suppressing interferon responses. Together, these findings highlight the critical role of waterfowl in ARV evolution and provide essential insights into the pathogenesis of emerging reoviruses. Moving forward, functional studies targeting the σC DE-loop and specific viral antagonists will be crucial to elucidate how these viruses balance receptor adaptability with host defense suppression to achieve successful host jumps. Nevertheless, this study has some limitations. First, while embryo and cell models provided preliminary evidence of cross-species infectivity, the viral dynamics in adult poultry under natural conditions require further validation. Additionally, the specific molecular functions of the σC DE-loop in host-range expansion warrant deeper investigation through site-directed mutagenesis in future studies.

## Data Availability

The data supporting the findings of this study are available from the corresponding author upon reasonable request. The genome sequence and transcriptomic data will be deposited in public databases upon acceptance of the manuscript.

## Supplementary Materials

The following supporting information can be downloaded from: https://www.mdpi.com/article/10.3390/v18040447/s1, Figure S1: Transcriptomic analysis and qRT-PCR validation of SD0407-infected GEF cells; Table S1: Primers used for the whole-genome amplification of the SD0407 strain; Table S2: Primers used for real-time quantitative PCR (RT-qPCR) analysis; Table S3: GenBank accession numbers of avian orthoreovirus genomes referenced in this study; Table S4: Genome organization and ORF annotation of goose-origin avian orthoreovirus strain SD0407 [20,28,29,30,31,32,33,34,35,36,37,38,39,40,41,42,43,44,45,46,65].
