# Supplementary material for: Characterization of a Goose-Origin Avian Orthoreovirus with Interferon Suppression Activity
Source: Viruses. 2026 Apr 8;18(4):447. doi: 10.3390/v18040447 (PMC13119739; doi:10.3390/v18040447)
Supplement: Supplementary file 1 [file viruses-18-00447-s001.zip › viruses-4145501-supplementary.pdf]

Supplmentary material

Supplment Figure S1

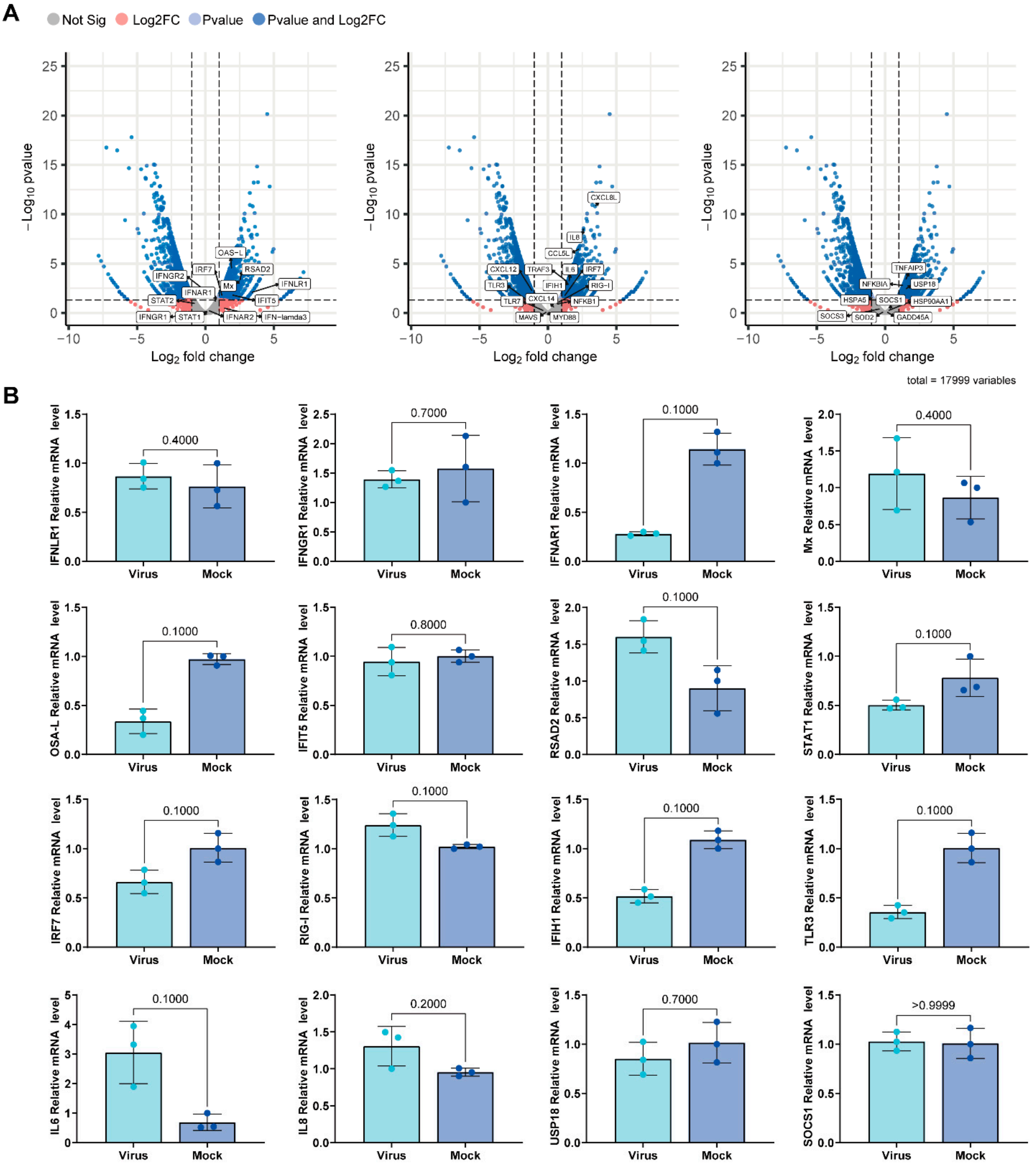

(A) Volcano plots showing differentially expressed genes (DEGs) between SD0407-infected and mock-infected cells at 24 h post infection ($|log_2FC| > 1$, $p < 0.05$). Representative immune- and interferon-related DEGs are labeled. (B) qRT-PCR validation of selected DEGs confirming the

RNA-seq data. Data are presented as mean ± SEM (n = 3). Significance was determined by Student’ s t-test ($p < 0.05$).

Supplment Table S1 primers used for amplying goose reovirus genome.

Genome	Forward primer (5′→3′)	Reverse primer(5′→3′)	Product size(bp)
L1	GCTTTTTCTCCGAACGCCG	GATGAATAACCTCCAACGAGAGTCG	3959
L2	GCTTTTTCCTCACCATGC	GATGAGTAATTCCTCGAGCCATGCT	3830
L3	GCTTTTACACCCATGGCTCA	GATGAGTAACACCCTTCTACTGGA	3907
M1	GCTTTTCTCGACATGGCCTATCTAG	GATGAATATCTCAAGACGGCTAACCCA	2283
M2	GCTTTTTCAGTGCCAGTCTTT	GATGAATAACGTGCCAATCCAG	2158
M3	GCTTTTTGAGTCCTAGCGTGG	GATGAGTAACCGAGTCCGCCG	1996
S1	GCTTTTTTCTTCTCTGCCCATGG	GATGAATAGCTCTTCTCATCGTGC	1568
S2	GCTTTTTCTCCCACGATGGCG	GATGAATACACCCACGCGCTAC	1324
S3	GCTTTTTGAGTCCTCAGCGTG	GATGAATAGGCGAGTCCCGC	1202
S4	GCTTTTTGAGTCCTTGTGCAGC	GATGAATAAGAGTCCAAGTCGCGG	1191

Supplment Table S2 Primers for RT-qPCR.

Primer name	sequence(5′-3′)	Primer name	sequence(5′-3′)
q-IFNAR1-F	AGTGGCTCAATGTACCGAGA	q-RSAD2-F	AAACTGAGACAGTGGTGCCG
q-IFNAR1-R	ATTGACTCATGGCCTGCACA	q-RSAD2-R	CACAGGGTTGAGTGCCATGA
q-IFNGR1-F	TCGGACAGTAACCAAACAGGT	q-RIG-I-F	GGAGAAGCATTCAAGGAGCG
q-IFNGR1-R	GGGGCTTGTCATAGCCAGAA	q-RIG-I-R	CTGTGATTCCCCAGTCATGC

q-IFNLR1-F	TGGCCTCTCTCCTGTCATCA	q-MDA5-F	GGCCTCGACATCAAAGAGTG
q-IFNLR1-R	GTTCTGGACGGCTGGTAGTT	q-MDA5-R	GCATAGGTGCTCTCATCAGC
q-STAT1-F	ACGCGCTCTATTGAAGGACC	q-TLR3-F	AGCTTGTGTTGTGTGGCAAT
q- STAT1-R	TGGGGCTCGTTTTGAGATCC	q-TLR3-R	TGCACGTAAACATCCTGTGTG
q-IL6-F	CACAAAGCATCTGGCAACGA	q-IRF7-F	GGAGACCCCCATCTTTGACT
q-IL6-R	ACTTCAGCTTTGTGAGGAGGG	q-IRF7-R	AGGAAGATGGTGAAGTCGGG
q-IL8-F	AGGACAACAGAGAGGTGTGC	q-USP18-F	GCTGCCCAACTCTTTCTGAC
q-IL8-R	CAGCGGTGCATCAGAATTGA	q- USP18-R	GGTGCTCCTGTACGCAGATA
q-Mx-F	ACGAGGCACACCCACAATAG	q-SOCS1-F	GCCCATGAGAAGCTGAAGTC
q-Mx-R	AGCAAGTGTTCCTCTCCACG	q-SOCS1-R	GCGCCCAGTCTGAAAGTTTA
q-OSAL-F	CCTGGTCAAGCACTGGTACA	q-IFIT5-F	AGCCTTAGCAGTGACACCAA
q-OSAL-R	CCACGCATAGATGGTCAGCA	q-IFIT5-R	GGTGGATTTCCTGCCCTTTG

Supplment Table S3 GenBank numbers of the genomes referenced in this article

**Organism Scientific Name**	**Organism Qualifier**	**Assembly Accession**	**Source**	**Reference**
Avian orthoreovirus	AVS-B	GCA_000891595.1	GenBank	[1]
Avian orthoreovirus	GX/2010/1	GCA_003092515.1	GenBank	[2]
Avian orthoreovirus	17203-M-06	GCA_003092535.1	GenBank	[3]
Avian orthoreovirus	19831M09	GCA_003092555.1	GenBank	[4]
Avian orthoreovirus	3211-V-02	GCA_003092575.1	GenBank	[3]
Avian orthoreovirus	526	GCA_003092595.1	GenBank	Unpublished
Muscovy duck reovirus	815-12	GCA_003092615.1	GenBank	[5]
Avian orthoreovirus	C78	GCA_003092635.1	GenBank	Unpublished
Avian orthoreovirus	HB10-1	GCA_003092655.1	GenBank	Unpublished
Muscovy duck reovirus	CA	GCA_003092675.1	GenBank	Unpublished
Avian orthoreovirus	D1007	GCA_003092695.1	GenBank	Unpublished
Avian orthoreovirus	D1104	GCA_003092715.1	GenBank	[4]

Avian orthoreovirus	D1246	GCA_003092735.1	GenBank	[4]
Avian orthoreovirus	D20/99	GCA_003092755.1	GenBank	[6]
Avian orthoreovirus	GX110058	GCA_003092775.1	GenBank	Unpublished
Avian orthoreovirus	GX110116	GCA_003092795.1	GenBank	Unpublished
Avian orthoreovirus	GuangxiR2	GCA_003092815.1	GenBank	Unpublished
Avian orthoreovirus	LN09-1	GCA_003092835.1	GenBank	Unpublished
Duck reovirus	HN5d	GCA_003092855.1	GenBank	Unpublished
Muscovy duck reovirus	J18	GCA_003092875.1	GenBank	[7]
Avian orthoreovirus	K738/14	GCA_003092895.1	GenBank	Unpublished
Avian orthoreovirus	Reo/PA/Broiler/05682/12	GCA_003092935.1	GenBank	[8]
Avian orthoreovirus	Reo/PA/Layer/27614/13	GCA_003092955.1	GenBank	Unpublished
Avian orthoreovirus	Reo/PA/Pheasant/13649/14	GCA_003092975.1	GenBank	Unpublished
Avian orthoreovirus	Reo/PA/Turkey/22342/13	GCA_003092995.1	GenBank	[9]
Duck reovirus SD-12	SD-12	GCA_003093015.1	GenBank	[10]
Avian orthoreovirus	T1781	GCA_003093035.1	GenBank	[11]
Duck reovirus	ZJ00M	GCA_003093055.1	GenBank	[12]
Muscovy duck reovirus	ZJ2000M	GCA_003093075.1	GenBank	[13]
Avian orthoreovirus	S1133	GCA_003093095.1	GenBank	Unpublished
Avian orthoreovirus	Reo/PA/Layer/01224A/14	GCA_003093115.1	GenBank	[14]
Avian orthoreovirus	Reo/PA/Layer/01224B/14	GCA_003093135.1	GenBank	[14]
Avian orthoreovirus	SD09-1	GCA_003093155.1	GenBank	Unpublished
Avian orthoreovirus	SD10-1	GCA_003093175.1	GenBank	Unpublished
Duck reovirus	091	GCA_003093195.1	GenBank	[15]
Avian orthoreovirus	S1133	GCA_013086095.1	GenBank	Unpublished
Goose orthoreovirus	03G	GCA_023156455.1	GenBank	[16]
Muscovy duck reovirus	S14	GCA_023182855.1	GenBank	[17,18]
Avian orthoreovirus	turkey reovirus TX99	GCA_026210815.1	GenBank	[19]
Goose reovirus	GRV-GD2020	GCA_031528155.1	GenBank	Unpublished
Duck reovirus	Ych	GCA_031528445.1	GenBank	[20]
Duck reovirus	NP03/CHN/2009	GCA_031528785.1	GenBank	[21]
Avian orthoreovirus	Pycno-1	GCF_003092915.1	RefSeq	Unpublished
Avian orthoreovirus	HB10-1	GCA_003092655.1	GenBank	Unpublished

Supplment Table S4 Genome organization and ORF annotation of goose-origin avian orthoreovirus strain SD0407.

Segment Group	Segment	Segment Length (bp)	ORF Position	Amino Acids (aa)	Encoded Protein
L	L1	3959	22···3903	1293aa	λA
	L2	3830	15···3794	1259aa	λB
	L3	3907	13···3870	1285aa	λC
M	M1	2283	13···2211	732aa	μA
	M2	2158	30···2057	675aa	μB
	M3	1996	25···1932	635aa	μNS
S	S1	1568	571···1536	321aa	σC
			20···313	97aa	P10
			273···761	162aa	P17
	S2	1324	16···1266	416aa	σA
	S3	1202	31···1134	367aa	σB
	S4	1191	24···1127	367aa	σNS

1. Bányai, K.; Dandár, E.; Dorsey, K.M.; Mató, T.; Palya, V. The genomic constellation of a novel avian orthoreovirus strain associated with runting-stunting syndrome in broilers. *Virus Genes* **2011**, *42*, 82-89, doi:10.1007/s11262-010-0550-z.
2. Li, C.; Wei, H.; Yu, L.; Duan, S.; Cheng, J.; Yan, W.; Zhang, X.; Wu, Y. Nuclear localization of the p17 protein of avian reovirus is correlated with autophagy induction and an increase in viral replication. *Arch Virol* **2015**, *160*, 3001-3010, doi:10.1007/s00705-015-2598-5.
3. Farkas, S.L.; Marton, S.; Dandár, E.; Kugler, R.; Gál, B.; Jakab, F.; Bálint, Á.; Kecskeméti, S.; Bányai, K. Lineage diversification, homo- and heterologous reassortment and recombination shape the evolution of chicken orthoreoviruses. *Sci Rep* **2016**, *6*, 36960, doi:10.1038/srep36960.
4. Dandár, E.; Fehér, E.; Bálint, Á.; Kisfali, P.; Melegh, B.; Mató, T.; Kecskeméti, S.; Palya, V.; Bányai, K.; Farkas, S.L. Genome Sequences of Three Turkey Orthoreovirus

Strains Isolated in Hungary. *Genome Announc* **2015**, *3*, doi:10.1128/genomeA.01333-15.
5. Wang, D.; Shi, J.; Yuan, Y.; Zheng, L.; Zhang, D. Complete sequence of a reovirus associated with necrotic focus formation in the liver and spleen of Muscovy ducklings. *Vet Microbiol* **2013**, *166*, 109-122, doi:10.1016/j.vetmic.2013.05.022.
6. Dandár, E.; Farkas, S.L.; Marton, S.; Oldal, M.; Jakab, F.; Mató, T.; Palya, V.; Bányai, K. The complete genome sequence of a European goose reovirus strain. *Arch Virol* **2014**, *159*, 2165-2169, doi:10.1007/s00705-014-2003-9.
7. Wang, D.; Xu, F.; Ma, G.; Zhang, C.; Huang, Y.; Li, H.; Zhang, D. Complete genomic sequence of a new Muscovy duck-origin reovirus from China. *J Virol* **2012**, *86*, 12445, doi:10.1128/jvi.02230-12.
8. Tang, Y.; Lu, H. Genomic characterization of a broiler reovirus field strain detected in Pennsylvania. *Infect Genet Evol* **2015**, *31*, 177-182, doi:10.1016/j.meegid.2015.01.029.
9. Tang, Y.; Lu, H.; Sebastian, A.; Yeh, Y.T.; Praul, C.A.; Albert, I.U.; Zheng, S.Y. Genomic characterization of a turkey reovirus field strain by Next-Generation Sequencing. *Infect Genet Evol* **2015**, *32*, 313-321, doi:10.1016/j.meegid.2015.03.029.
10. Yu, K.; Li, Y.; Han, H.; Song, M.; Ma, X.; Liu, C.; Huang, B.; Li, F. Complete genome sequence of an avian reovirus isolated from wild mallard ducks in china. *Genome Announc* **2014**, *2*, doi:10.1128/genomeA.00813-14.
11. Dandár, E.; Bálint, A.; Kecskeméti, S.; Szentpáli-Gavallér, K.; Kisfali, P.; Melegh, B.; Farkas, S.L.; Bányai, K. Detection and characterization of a divergent avian reovirus strain from a broiler chicken with central nervous system disease. *Arch Virol* **2013**, *158*, 2583-2588, doi:10.1007/s00705-013-1739-y.
12. Yun, T.; Yu, B.; Ni, Z.; Ye, W.; Chen, L.; Hua, J.; Zhang, C. Genomic characteristics of a novel reovirus from Muscovy duckling in China. *Vet Microbiol* **2014**, *168*, 261-271, doi:10.1016/j.vetmic.2013.11.005.
13. Yun, T.; Yu, B.; Ni, Z.; Ye, W.; Chen, L.; Hua, J.; Zhang, C. Isolation and genomic characterization of a classical Muscovy duck reovirus isolated in Zhejiang, China. *Infect Genet Evol* **2013**, *20*, 444-453, doi:10.1016/j.meegid.2013.10.004.
14. Tang, Y.; Lin, L.; Sebastian, A.; Lu, H. Detection and characterization of two co-infection variant strains of avian orthoreovirus (ARV) in young layer chickens using next-generation sequencing (NGS). *Sci Rep* **2016**, *6*, 24519, doi:10.1038/srep24519.
15. Ma, G.; Wang, D.; Shi, J.; Jiang, T.; Yuan, Y.; Zhang, D. Complete genomic sequence of a reovirus isolate from Pekin ducklings in China. *J Virol* **2012**, *86*, 13137, doi:10.1128/jvi.02512-12.
16. Yun, T.; Ye, W.; Ni, Z.; Chen, L.; Yu, B.; Hua, J.; Zhang, Y.; Zhang, C. Complete genomic sequence of goose-origin reovirus from China. *J Virol* **2012**, *86*, 10257, doi:10.1128/jvi.01692-12.
17. Zhang, Y.; Liu, M.; Shuidong, O.; Hu, Q.L.; Guo, D.C.; Chen, H.Y.; Han, Z. Detection and identification of avian, duck, and goose reoviruses by RT-PCR: goose and duck reoviruses are part of the same genogroup in the genus Orthoreovirus. *Arch Virol* **2006**, *151*, 1525-1538, doi:10.1007/s00705-006-0731-1.
18. Zhang, Y.; Guo, D.; Geng, H.; Liu, M.; Hu, Q.; Wang, J.; Tong, G.; Kong, X.; Liu, N.; Liu, C. Characterization of M-class genome segments of muscovy duck reovirus S14. *Virus Res* **2007**, *125*, 42-53, doi:10.1016/j.virusres.2006.12.004.
19. Sellers, H.S.; Linnemann, E.G.; Pereira, L.; Kapczynski, D.R. Phylogenetic analysis of the sigma 2 protein gene of turkey reoviruses. *Avian Dis* **2004**, *48*, 651-657,

doi:10.1637/7181-032304r.
20. Cao, Y.; Sun, M.; Wang, J.; Hu, X.; He, W.; Su, J. Phenotypic and genetic characterisation of an emerging reovirus from Pekin ducks in China. *Sci Rep* **2019**, *9*, 7784, doi:10.1038/s41598-019-44178-3.
21. Chen, S.Y.; Chen, S.L.; Lin, F.Q.; Wang, S.; Jiang, B.; Cheng, X.X.; Zhu, X.L.; Li, Z.L. [The isolation and identification of novel duck reovirus]. *Bing Du Xue Bao* **2012**, *28*, 224-230.
